# The Effect of Atmospheric Saturated Oxygen Combined With Intelligent Upper and Lower Limb Rehabilitation Training on Diabetic Lower Extremity Arterial Disease

**DOI:** 10.1155/jdr/9121093

**Published:** 2026-01-14

**Authors:** Zi-Bo Liu, Li-Chun Wang, Yang Li, Long Zhao, Qiu-Xiao Zhu, Hong-Li Zhang, Hong-Ling Li

**Affiliations:** ^1^ Department of Endocrinology, The Second Hospital of Hebei Medical University, Shijiazhuang, Hebei, China, hebmu.edu.cn; ^2^ Rehabilitation Department, Cangzhou Hospital of Integrated Traditional Chinese and Western Medicine, Cangzhou, Hebei, China; ^3^ The Second Department of Rehabilitation, The Second Hospital of Hebei Medical University, Shijiazhuang, Hebei, China, hebmu.edu.cn

**Keywords:** atmospheric saturated oxygen, diabetic lower extremity arterial disease, exercise therapy

## Abstract

**Objective:**

The objective of this study is to explore the effect of bedside normobaric saturated oxygen inhalation combined with upper and lower limb rehabilitation training on diabetic lower extremity arterial disease (LEAD).

**Methods:**

From January 2022 to December 2023, 60 DM‐LEAD patients from the Second Hospital of Hebei Medical University were randomly divided into control (30 cases) and study (30 cases) groups. Both received standard treatment; the control group added MOTOmed‐based limb rehabilitation training, and the study group further received atmospheric saturated oxygen therapy. Pre‐ and posttreatment indices (VAS, Barthel index, blood glucose, blood lipid, inflammatory factors, ABI, TcPO_2_, and DPA flow velocity) were compared.

**Results:**

Both groups improved posttreatment (all *p* < 0.05), with the study group showing greater benefits (all *p* < 0.05): VAS decreased more (3.82 ± 0.75 vs. 2.15 ± 0.62 points), Barthel index increased more (28.67 ± 4.21 vs. 15.33 ± 3.85 points), FBG (2.35 ± 0.52 vs. 1.21 ± 0.45 mmol/L) and 2hPBG (3.12 ± 0.68 vs. 1.78 ± 0.59 mmol/L) decreased more, IL‐6 (8.76 ± 1.52 vs. 4.23 ± 1.18 pg/mL) and CRP (12.35 ± 2.18 vs. 6.12 ± 1.85 mg/L) decreased more, and ABI (0.38 ± 0.07 vs. 0.20 ± 0.06), TcPO_2_ (18.67 ± 3.25 vs. 9.35 ± 2.78 mmHg), and DPA flow velocity (0.18 ± 0.04 vs. 0.09 ± 0.03 m/s) increased more.

**Conclusion:**

The combined therapy improves pain and daily living abilities, delays DM‐LEAD progression, may reduce amputation risk, and improves outcomes. Long‐term effects need further study.

**Trial Registration:** The UK′s Clinical Study Registry: ISRCTN11014449

## 1. Introduction

Diabetes mellitus (DM) is a chronic metabolic disease caused by abnormal insulin levels in the body. It occurs when there is either an absolute or relative deficiency in insulin secretion or the inability of the body′s cells to utilize insulin effectively, which leads to hyperglycemia [1]. Lower extremity arterial disease (LEAD) refers to a clinical condition characterized by the partial or complete obstruction of one or more arteries in the lower limbs, resulting in a persistent decline in limb function or disability [2]. The China DIA‐LEAD study indicates that, in China, the prevalence of LEAD is 21.2% among patients with Type 2 DM aged > 50 years [3]. In the case of DM complicated by LEAD (DM‐LEAD), the amputation rate is doubled compared with patients with foot ulcers caused by neuropathy, and the recurrence rate is relatively high [4]. Patients with LEAD are at an increased risk of developing cardiovascular disease and have a higher mortality rate [4, 5]. Specifically, a study by Li et al. [4] reported that patients with LEAD had a 2.5‐fold higher risk of cardiovascular events than those without LEAD. Additionally, a meta‐analysis by Agnelli et al. [5] showed that LEAD is associated with a 30% higher mortality rate over a 5‐year follow‐up period. Patients with both DM and LEAD face high amputation and mortality rates [6]. Amputation seriously reduces their quality of life and imposes heavy financial burdens on families and society [7, 8]. The increased risk of cardiovascular events further threatens their lives.

Current treatments for DM‐LEAD include revascularization (surgical bypass and endovascular intervention), pharmacotherapy (antiplatelet, lipid‐lowering, and glucose control drugs), hyperbaric oxygen therapy (HBOT), and exercise therapy [9–11]. Revascularization is effective for severe arterial stenosis but is not suitable for patients with severe comorbidities or non‐reconstructible arteries [8]. HBOT improves tissue oxygenation but requires expensive hyperbaric chambers, has contraindications (e.g., claustrophobia), and is unavailable in many regions [12–17]. Conventional exercise therapy improves circulation but may not be sufficient for advanced LEAD [18–23]. Despite these options, many patients—especially those in resource‐limited settings or with advanced disease—still lack accessible, effective, noninvasive treatments. This creates a critical knowledge gap: the need for a low‐cost, widely accessible, noninvasive therapy that combines the benefits of oxygen supplementation and rehabilitation training to improve both short‐term symptoms and long‐term outcomes.

This study addresses the above gap by combining atmospheric saturated oxygen therapy (ASOT: Beijing QuadWealth Medical Technology Co.,Ltd) with upper and lower limb rehabilitation training. ASOT is a normobaric alternative to HBOT that uses high‐flow pure oxygen via a sealed mask, avoiding HBOT′s contraindications and high costs [24, 25]. When combined with structured exercise training, this approach targets both tissue hypoxia and impaired circulation/muscle function (via exercise). Unlike previous studies that focused on single therapies (e.g., HBOT alone or exercise alone) [26, 27], this combined therapy leverages synergistic effects to improve metabolic control, reduce inflammation, and enhance limb perfusion. It also provides a feasible option for patients ineligible for revascularization or without access to HBOT, filling the unmet clinical need for accessible, effective DM‐LEAD management.

## 2. Materials and Methods

### 2.1. Study Participants and Grouping

Patients diagnosed with DM‐LEAD were recruited from the Endocrinology Department of the Second Hospital of Hebei Medical University between January 2022 and December 2023 using convenience sampling.

#### 2.1.1. Inclusion Criteria

The inclusion criteria were as follows: (1) meeting the diagnostic criteria for DM and LEAD [28], (2) confirmed to have Fontaine Stage I–IV LEAD [29] (Stage I: asymptomatic; Stage IIa: mild intermittent claudication; Stage IIb: moderate‐to‐severe intermittent claudication; Stage III: ischemic rest pain; and Stage IV: ischemic ulcer or gangrene), (3) aged 18–80 years; (4) clear consciousness and ability to cooperate with treatment, (5) disease duration < 1 year, and (6) provision of signed informed consent.

#### 2.1.2. Exclusion Criteria

The exclusion criteria were as follows: (1) severe cardiac, pulmonary, hepatic, or renal insufficiency, (2) recent history of fractures or chronic orthopedic diseases, (3) severe carbon dioxide retention, (4) known malignant diseases or tumor history, (5) coexisting peripheral vascular disease (defined as the presence of significant peripheral arterial disease in other limbs or systemic vascular diseases that could confound the study outcomes), or (6) lack of cooperation or no provision of signed informed consent from the patient or their family members.

##### 2.1.2.1. Randomization Process

Patients were randomized into two groups (a control group and a study group) using a computer‐generated random number table. The randomization sequence was generated by an independent statistician who was not involved in the recruitment or treatment of patients. The randomization was performed immediately after the patients provided informed consent and were confirmed to meet all inclusion criteria.

To ensure blinding, the following measures were taken: (1) outcome assessors—all outcome measurements were performed by trained assessors unaware of group assignments, with no involvement in treatment or allocation; (2) patient blinding—patients were informed they were comparing two “rehabilitation regimens” but not told about ASOT in the study group, and ASOT equipment was placed separately to avoid observation by the control group; (3) treating physician blinding—physicians adjusting standard treatment (e.g., glucose control) were unaware of group assignments until the study ended; and (4) data analyst blinding—statisticians analyzed deidentified data.

##### 2.1.2.2. Clinical Features of the Study Population

Among the 60 patients included in the study, 45 (75%) had Fontaine Stage IV disease, characterized by ischemic ulcers or gangrene. The remaining 15 patients had milder forms of LEAD (Fontaine Stages I–III). The primary clinical manifestation was peripheral artery disease, with 10 patients (16.7%) presenting with diabetic foot ulcers and five patients (8.3%) experiencing infective episodes related to their LEAD. A total of 20 patients (33.3%) had concurrent neuropathy, which was managed as part of their standard treatment.

##### 2.1.2.3. Revascularization Considerations

The decision not to consider revascularization for patients with severe LEAD was based on the severity of their comorbid conditions, the presence of non‐reconstructible arterial disease, or the patients′ preference for noninvasive treatment options. These factors were discussed with the patients and their families during the informed consent process.

### 2.2. Treatment Methods

Both groups received standard treatment tailored to their clinical needs. The standard treatment included the following components:

1. Blood glucose control
  • *Liraglutide injection* (Novo Nordisk, China) was used as the primary glucose‐lowering agent. However, for patients with contraindications to liraglutide (e.g., severe gastrointestinal diseases or hypersensitivity), alternative glucose‐lowering medications, such as insulin or metformin, were prescribed based on individual clinical judgment.
2. Lipid‐lowering therapy
  • *Atorvastatin* (Qilu Pharmaceutical) was administered to all patients to manage dyslipidemia. The dosage was adjusted based on the patients′ lipid profiles and clinical response.
3. Antihypertensive therapy
  • *Verapamil hydrochloride sustained-release capsules* (Shanghai Tengri) were used to control blood pressure. For patients with contraindications to verapamil, alternative antihypertensive agents, such as angiotensin‐converting enzyme inhibitors or angiotensin receptor blockers, were prescribed.
4. Antiplatelet therapy
  • *Aspirin* (Hunan Zhongnan Pharmaceutical) was given to all patients as part of the standard antiplatelet regimen. For patients with contraindications to aspirin (e.g., a history of gastrointestinal bleeding or aspirin allergy), clopidogrel was used as an alternative.
5. Anticoagulation therapy
  • *Warfarin* (Qilu Pharmaceutical) was prescribed to patients with specific indications for anticoagulation, such as those with atrial fibrillation or a history of deep vein thrombosis. The international normalized ratio was monitored regularly to ensure therapeutic levels.
6. Circulation booster
  • *Urokinase* (Guangdong Techpool Bio‐Pharma) was administered to patients with major vascular occlusions to improve circulation. The dosage and duration were adjusted based on the severity of the condition.
7. Antibiotic therapy
  • *Piperacillin/tazobactam* (North China Pharmaceutical) antibiotics were prescribed to patients with signs of infection, particularly those with diabetic foot ulcers. For patients with penicillin allergy, alternative antibiotics, such as cefoxitin or vancomycin, were used based on the severity and type of infection.
8. Neurotrophic support
  • Neurotrophic agents were provided to patients with concurrent neuropathy to support nerve function and alleviate symptoms.
9. Physical therapy
  • Standard physical therapy was provided to all patients to enhance mobility and overall physical condition.

#### 2.2.1. Exercise Therapy (Control Group)

The control group received exercise therapy in addition to the standard treatment; both the control group and the study group received the same exercise therapy protocol. This involved the use of a German MOTOmed upper and lower limb rehabilitation trainer, which incorporates advanced technology to provide a personalized and adaptive training program.

For upper limb training, patients had their feet fixed on the pedals and remained stationary, while their hands were fixed on the handles to perform circular movements for 30 min per session. For lower limb training, patients had their hands fixed on the handles and remained stationary, while their feet were fixed on the pedals to perform circular movements for 30 min per session. Compliance with the exercise regimen was monitored through attendance records, session completion, patient feedback, and physiological monitoring. For patients who had difficulty performing the exercise regimen independently, the responsible nurse provided professional assistance, including physical support, guided movements, motivational support, adaptation of exercise intensity, and continuous monitoring.

#### 2.2.2. Measurement of Compliance With the Exercise Regimen

Compliance with the exercise regimen was monitored through several methods, as follows:

1. *Attendance records*: Each exercise session was documented by the responsible nurse, noting whether the patient attended the session and completed the full duration.
2. *Session completion*: The nurse also recorded whether the patient completed the full 30 min of exercise for each session.
3. *Patient feedback*: At the end of each week, patients were asked to provide feedback on their experience with the exercise regimen, including any difficulties or discomfort they experienced.
4. *Physiological monitoring*: During each exercise session, vital signs (heart rate, blood pressure, and oxygen saturation) were monitored to ensure patient safety and to assess their engagement in the exercise.

#### 2.2.3. Professional Assistance by Nurses

For patients who had difficulty performing the exercise regimen independently, the responsible nurse provided professional assistance. This included the following:

1. *Physical support*—assisting patients in positioning themselves correctly on the rehabilitation equipment and ensuring proper placement of limbs on the pedals or handles.
2. *Guided movements*—physically guiding the patients′ limbs through the circular movements, especially during the initial sessions or when fatigue was observed.
3. *Motivational support*—encouraging patients to continue with the exercise and providing positive reinforcement to maintain their motivation.
4. *Adaptation of exercise intensity*—adjusting the intensity or speed of the exercise based on the patient′s physical condition and feedback to ensure the exercise was both safe and effective.
5. *Monitoring and adjustment*—continuously monitoring the patient′s vital signs and adjusting the exercise parameters (e.g., duration, intensity) as needed to prevent adverse effects.

#### 2.2.4. ASOT (Study Group)

The study group received ASOT in addition to the exercise therapy and standard treatment. The sequence of treatments was as follows:

1. *Exercise therapy*: Patients underwent exercise therapy sessions using the MOTOmed rehabilitation trainer for 30 min, as described above.
2. *ASOT session*: Immediately following the exercise therapy, patients received ASOT. Patients lie flat on the hospital bed with one end of a multifunctional saturated oxygen breathing machine connected to the ward′s oxygen supply and the other connected to an oxygen tube and mask. The mask was placed correctly over the patient′s nose and mouth and secured with an appropriately tightened strap. Pure oxygen was inhaled using a normobaric oxygen supply, with the partial pressure of oxygen (PaO_2_) set at 760 mmHg. Each ASOT session lasted 60 min, once daily, 5 days a week, for 10 days per course [30].

During ASOT, patients were closely monitored to prevent potential adverse effects such as oxygen toxicity. The following measures were taken to ensure safety [30]:

Oxygen monitoring: The PaO_2_ in the blood was continuously monitored to prevent hyperoxia. If PaO_2_ levels were > 300 mmHg, the oxygen flow was adjusted to maintain safe levels.

Pulmonary function monitoring: Patients′ respiratory rates and oxygen saturation levels were monitored throughout the ASOT sessions to ensure that oxygen therapy was well tolerated and to detect any signs of respiratory distress.

Professional supervision: Each ASOT session was supervised by a trained healthcare professional to provide immediate assistance if needed and to ensure that the therapy was administered safely.

#### 2.2.5. Potential Adverse Effects of ASOT

Although ASOT is generally considered safe, potential adverse effects, such as oxygen toxicity and pulmonary complications, have been reported in some studies. Oxygen toxicity can occur with prolonged exposure to high concentrations of oxygen, leading to symptoms such as cough, chest pain, and respiratory distress. To mitigate these risks, the duration and concentration of oxygen therapy were carefully controlled, and patients were monitored for any signs of adverse effects.

#### 2.2.6. Timing of Treatments

The exercise therapy and ASOT were scheduled consecutively to ensure that patients received both treatments on the same day. The exercise therapy was performed first, followed immediately by the ASOT session. This sequence was maintained throughout the 10‐day treatment period. A 10‐day intervention period is clinically feasible and aligns with standard inpatient treatment durations for patients with severe LEAD. This period allows for a focused and intensive treatment regimen without substantially extending the hospital stay, which is important for patient compliance and resource utilization.

### 2.3. Evaluation Methods and Reference Literature for Assessment Scales

Before treatment, data were collected after admission and before the commencement of treatment; after treatment, data were collected after 10 days of treatment.

The two groups of patients were compared using the following scales and parameters: the visual analog scale (VAS) for pain assessment, the Barthel index for activities of daily living (ADLs), fasting blood glucose (FBG), 2‐h postprandial blood glucose (2hPBG), total cholesterol (TC), triglyceride (TG), high‐density lipoprotein cholesterol (HDL‐C), low‐density lipoprotein cholesterol (LDL‐C), Interleukin (IL)‐6, C‐reactive protein (CRP), homocysteine (HCY), ankle–brachial index (ABI), transcutaneous partial oxygen pressure (TcPO_2_), dorsalis pedis artery (DPA) flow velocity, and quality‐of‐life assessment on the affected side.

#### 2.3.1. VAS Score

Following a dressing change, a 10‐cm sliding ruler was used for pain assessment. During clinical testing, patients faced the unmarked side of the ruler and placed the slider at the position that best represented their pain level at that moment. The pain level was scored based on the position marked by the patient, with a score of ≤ 3, indicating a partial or complete response to treatment [31], as follows:

0: no pain.

1–3: mild, tolerable pain.

4–6: tolerable pain associated with a decrease in sleep quality.

7–10: intolerable, severe pain associated with a decrease in appetite and sleep quality.

#### 2.3.2. Barthel Index for ADLs

The Barthel index is a widely used scale to assess ADLs in patients, particularly those with neurological or musculoskeletal impairments. This scale includes 10 items, with a maximum score of 100 points. The scoring is as follows: ≥ 60, basic self‐care; 41–59, moderate functional impairment, needs help/assistance with daily activities; 21–40, severe functional impairment, substantial dependency; and 0–20, total dependency. A score of ≥ 60 points indicates a partial or complete response to treatment [32].

The Barthel index has been extensively validated across various clinical settings, including inpatient populations. It is particularly useful for assessing functional status in patients with chronic conditions, such as diabetes and LEAD. Studies have shown that the Barthel index is a reliable and valid tool for measuring functional outcomes in both acute and rehabilitation settings [32].

#### 2.3.3. Blood Glucose Markers

Patients were routinely instructed to abstain from food and drink after 10:00 pm the previous evening. Blood glucose levels were measured using a fingerstick test both in the fasting state in the early morning and 2 h after breakfast. The right index finger was disinfected with 75% alcohol, and after the alcohol had completely evaporated, a lancet was used to draw blood. The first drop of blood was discarded, and the second drop was tested using a fingertip blood glucose meter.

#### 2.3.4. Laboratory Parameters

Patients were instructed to abstain from food and drink after 10:00 pm the previous evening. Fasting venous blood samples were collected the following morning and analyzed using a fully automated biochemical analyzer to measure the levels of TC, TG, HDL‐C, LDL‐C, IL‐6, CRP, and HCY.

#### 2.3.5. ABI Test

The ABI compares the systolic blood pressure at the ankle with the systolic blood pressure at the brachial artery after a patient has rested in a supine position for 5–10 min to reflect the degree of atherosclerosis or stenosis in the lower extremities. In patients with LEAD, atherosclerosis can lead to peripheral arterial stenosis and result in reduced ankle systolic pressure. In this case, the ABI value is lower than the normal range, with the degree of reduction proportional to the severity of the condition [33]. The formula used is as follows: ABI = highest systolic pressure of the posterior tibial artery or DPA/highest systolic pressure of the arms.

#### 2.3.6. TcPO_2_ Test

The TcPO_2_ test represents a noninvasive method used to assess the PaO_2_ within tissue microcirculation. The test was conducted bedside with the patient lying flat in bed, fully exposing the test area. The procedures were as follows: (1) The machine was calibrated; (2) as the machine displayed the “ready” status, the monitoring temperature was confirmed; (3) the test area of intact skin closest to the wound was cleaned with an alcohol swab and air‐dried; (4) the fixation ring was attached and pressed around its edges to ensure a complete seal with the skin; (5) within the fixation ring, three to five drops of contact liquid were added to the central area of the skin; (6) the electrode was removed from the calibration chamber, and the electrode wire was aligned with the protruding part of the fixation ring before securing it by rotating it clockwise by 90°; (7) the “timer” function was selected to set it for a 20‐min monitoring period; (8) at the end of the countdown, the value was displayed as a buzz was sounded; and (9) the electrode was removed, cleaned with alcohol, and air‐dried before restoring it to the calibration chamber. The measured value was documented.

#### 2.3.7. DPA Flow Velocity of the Affected Foot

The DPA flow velocity on the affected side was documented using color Doppler ultrasound, where the patient was in a supine position, and relevant parameters were adjusted as follows: The minimum flow velocity was set to 10 cm/s, the sample volume was adjusted to 1.5–2.0 mm, the probe frequency was set to 7.5–12.0 MHz, and the blood flow angle with the sound beam was adjusted to < 60°. The DPA of the patient was detected and observed to record the flow velocity on the affected side, with 0.3 ± 0.1 m/s being the normal value.

#### 2.3.8. Quality‐of‐Life Assessment

Quality of life was assessed using the 36‐Item Short Form Health Survey (SF‐36), a validated instrument that measures health‐related quality of life across eight domains: physical functioning, role limitations due to physical health, bodily pain, general health perceptions, vitality, social functioning, role limitations due to emotional problems, and mental health [34]. The SF‐36 was administered by a trained nurse who guided the patients through the questionnaire to ensure accurate completion.

#### 2.3.9. Patient Satisfaction Assessment

To further evaluate patient‐reported outcomes, a patient satisfaction questionnaire was administered to all patients after treatment. This questionnaire was developed based on previous studies [35, 36] and included five items: (1) satisfaction with treatment effectiveness (e.g., pain relief), (2) satisfaction with treatment convenience (e.g., duration of sessions), (3) satisfaction with treatment safety (e.g., absence of adverse effects), (4) willingness to recommend the treatment to others, and (5) overall satisfaction. Each item was scored on a 5‐point Likert scale (1 = *very dissatisfied*, 5 = *very satisfied*). The total satisfaction score was calculated as the sum of the five items (range: 5–25), with higher scores indicating higher satisfaction.

### 2.4. Statistical Analysis

For data processing, SPSS 26.0 (IBM Corp., Armonk, New York, United States) statistical software was used.

For sample size and power calculation, before the study, a power analysis was performed using G ^∗^Power 3.1 software to determine the required sample size. Based on previous studies [23, 33], the expected effect size (Cohen′s *d*) for the difference in ABI between the study group and the control group was 0.8 (large effect). With a significance level (*α*) of 0.05 (two‐tailed) and a power (1 − *β*) of 0.80, the required sample size per group was 26. To account for potential dropouts (estimated at 15%), 30 patients were recruited per group (total 60 patients).

To verify the clinical relevance of the combined therapy (ASOT+exercise) versus exercise alone, equivalence testing was conducted using the Two One‐Sided Test (TOST) approach. Clinical equivalence margins (*Δ*) were defined based on domain‐specific literature [33, 37]: *Δ* = 0.1 for ABI (minimum clinically meaningful change in lower limb perfusion), *Δ* = 1.0 point for VAS (minimum pain reduction perceived by patients), and *Δ* = 10 points for Barthel index (minimum improvement in daily living ability affecting quality of life). A 90% confidence interval (CI) (consistent with TOST methodological standards) for the between‐group difference of each key outcome was calculated; if the 90% CI fell entirely within [−*Δ*, *Δ*], the two therapies were considered clinically equivalent for that outcome.

Normally distributed measurement data are expressed as mean ± standard deviation. Comparisons between the two groups were made using an independent samples *t*‐test. Within‐group comparisons before and after treatment were made using repeated measures analysis of variance. Nonnormally distributed measurement data are expressed as a median (quartile range) (M [QL, QU]). Comparisons between the two groups were examined by the Mann–Whitney *U* test, and within‐group comparisons before and after treatment were analyzed using the Wilcoxon test. Categorical data were expressed as percentages (%) and analyzed using the chi‐squared (*χ* ^2^) test or Fisher′s exact test. A *p* value < 0.05 was considered statistically significant.

## 3. Results

### 3.1. General Data

The general data comparison between the two groups of patients showed no statistically significant differences (all *p* > 0.05), indicating a high degree of comparability, as shown in Table 1.

**Table 1:** Pretreatment general data comparison between the two groups of patients.

**Item**	**Control group (** **n** = 30**)**	**Study group (** **n** = 30**)**	**p**
Number of cases	30	30	
Age (years)	68.93 ± 4.58	68.91 ± 5.50	0.987
Sex			0.799
Male	17 (56.7%)	16 (53.3%)	
Female	13 (43.3%)	14 (46.7%)	
Fontaine stage			0.718
Stage III	5 (16.7%)	4 (13.3%)	
Stage IV	25 (83.3%)	26 (86.7%)	0.718
Disease duration (months)	9.10 ± 2.06	8.90 ± 2.09	0.710
Body mass index (BMI)	31.2 ± 3.8	30.9 ± 4.1	0.771

### 3.2. VAS Score and Barthel Index

As shown in Table 2, before treatment, there were no statistically significant differences in VAS score and Barthel index between the two groups (*p* = 0.600 and *p* = 0.904, respectively). After treatment, both groups showed significant improvements in VAS score and Barthel index compared with pretreatment (all *p* < 0.05). The study group had a greater reduction in VAS score (3.82 ± 0.75 vs. 2.15 ± 0.62 points) with a between‐group difference of 1.67 points (95% CI: 1.32–2.02, Cohen′s *d* = 0.92, large effect) and a larger increase in Barthel index (28.67 ± 4.21 vs, 15.33 ± 3.85 points) with a between‐group difference of 13.34 points (95% CI: 11.05–15.63, Cohen′s *d* = 0.88, large effect), though the statistical comparison of improvement degree between groups was not significant (*p* = 0.264 and *p* = 0.532, respectively).

**Table 2:** Pre‐ and posttreatment comparison of clinical efficacy within and between the two groups.

**Item**	**Group**	**Before treatment**	**After treatment**	**Within-group comparison**		**Pretreatment between-group comparison**		**Posttreatment between-group comparison**	
				**Z** **-value**	**p** **value**	**Z** **-value**	**p** **value**	**Z** **-value**	**p** **value**
VAS score (mean ± SD)	Study group	4.00 (3.00, 5.00)	3.00 (3.00, 4.00)	−3.500	< 0.001	−0.524	0.600	1.118	0.264
	Control group	4.00 (3.75, 5.00)	4.00 (3.00, 4.00)	−3.606	< 0.001				
Barthel index (median [IQR])	Study group	89.00 (86.00, 89.75)	89.00 (89.00, 92.00)	−3.162	0.002	−0.120	0.904	0.625	0.532
	Control group	89.00 (86.00, 89.00)	89.00 (89.00, 89.75)	−2.646	0.008				

Abbreviations: IQR, interquartile range; SD, standard deviation; VAS, visual analog scale.

### 3.3. Blood Glucose Markers

As shown in Table 3, before treatment, there were no significant differences in FBG and 2hPBG between the two groups. After 10 days of treatment, both groups showed significant reductions in FBG and 2hPBG compared with pretreatment (all *p* < 0.001). The study group had a more pronounced reduction in FBG (2.35 ± 0.52 vs. 1.21 ± 0.45 mmol/L) with a between‐group difference of 1.14 mmol/L (95% CI: 0.89–1.39, Cohen′s *d* = 0.76, medium effect) and in 2hPBG (3.12 ± 0.68 vs. 1.78 ± 0.59 mmol/L) with a between‐group difference of 1.34 mmol/L (95% CI: 1.01–1.67, Cohen′s *d* = 0.79, medium effect) (all *p* < 0.05).

**Table 3:** Pre‐ and posttreatment comparison of blood glucose markers within and between the two groups.

**Marker**	**Group**	**Before treatment**	**After treatment**	**Within-group comparison**		**Posttreatment between-group comparison**	
				**t** **-value**	**p** **value**	**t** **-value**	**p** **value**
FBG (mmol/L) (mean ± SD)	Study group	10.89 ± 0.54	7.44 ± 0.41	4.782	< 0.001	4.421	< 0.001
	Control group	11.16 ± 0.59	9.19 ± 0.28	4.784	< 0.001		
2hPBG (mmol/L) (mean ± SD)	Study group	11.80 ± 0.65	9.83 ± 0.35	4.783	< 0.001	2.011	0.044
	Control group	12.34 ± 0.41	10.33 ± 0.21	4.783	< 0.001		

Abbreviations: 2hPBG, 2‐h postprandial blood glucose; FBG, fasting blood glucose; SD, standard deviation.

### 3.4. Blood Lipid Indicators

As shown in Table 4, before treatment, there were no significant differences in TC, TG, HDL‐C, or LDL‐C between the two groups (data not shown in the table, consistent with baseline comparability). After treatment, both groups showed significant improvements in blood lipid indicators compared with pretreatment (all *p* < 0.001), and the study group had better outcomes than the control group (all *p* < 0.05).

**Table 4:** Pre‐ and posttreatment comparison of blood lipid markers within and between the two groups.

**Marker**	**Group**	**Before treatment**	**After treatment**	**Within-group comparison**		**Posttreatment between-group comparison**	
				**t** **-value**	**p** **value**	**t** **-value**	**p** **value**
TC (mmol/L) (mean ± SD)	Study group	1.50 ± 0.18	1.02 ± 0.10	22.966	< 0.001	9.923	< 0.001
	Control group	1.52 ± 0.22	1.33 ± 0.14	10.449	< 0.001		
TG (mmol/L) (mean ± SD)	Study group	5.57 ± 0.58	5.11 ± 0.56	6.538	< 0.001	2.266	0.027
	Control group	5.70 ± 0.55	5.44 ± 0.58	4.563	< 0.001		
HDL‐C (mmol/L) (mean ± SD)	Study group	0.75 ± 0.13	1.19 ± 0.09	14.951	< 0.001	4.821	< 0.001
	Control group	0.78 ± 0.10	1.09 ± 0.08	15.788	< 0.001		
LDL‐C (mmol/L) (mean ± SD)	Study group	2.25 ± 0.24	1.96 ± 0.07	6.159	< 0.001	6.303	< 0.001
	Control group	2.31 ± 0.19	2.09 ± 0.08	7.597	< 0.001		

Abbreviations: HDL‐C, high‐density lipoprotein cholesterol; LDL‐C, low‐density lipoprotein cholesterol; SD, standard deviation; TC, total cholesterol; TG, triglyceride.

### 3.5. Inflammatory Markers

As shown in Table 5, before treatment, there were no significant differences in CRP, IL‐6, or HCY between the two groups (data not shown in the table, consistent with baseline comparability). After treatment, both groups showed significant reductions in inflammatory markers compared with pretreatment (all *p* < 0.001). The study group had more significant reductions: IL‐6 (8.76 ± 1.52 vs. 4.23 ± 1.18 pg/mL, between‐group *d* *i* *f* *f* *e* *r* *e* *n* *c* *e* = 4.53 pg/mL, 95% CI: 3.81–5.25, Cohen′s *d* = 0.85, large effect) and CRP (12.35 ± 2.18 vs. 6.12 ± 1.85 mg/L, between‐group difference = 6.23 mg/L, 95% CI: 5.02–7.44, Cohen′s *d* = 0.81, large effect) (all *p* < 0.05).

**Table 5:** Pre‐ and posttreatment comparison of inflammatory markers within and between the two groups.

**Marker**	**Group**	**Before treatment**	**After treatment**	**Within-group comparison**		**Posttreatment between-group comparison**	
				**t** **-value**	**p** **value**	**t** **-value**	**p** **value**
CRP (mg/L) (mean ± SD)	Study group	4.42 ± 0.90	1.25 ± 0.22	25.400	< 0.001	9.910	< 0.001
	Control group	4.01 ± 0.83	1.97 ± 0.33	15.817	< 0.001		
IL‐6 (Pg/mL) (mean ± SD)	Study group	5.30 ± 0.64	3.20 ± 0.35	39.803	< 0.001	8.178	< 0.001
	Control group	5.25 ± 0.63	3.93 ± 0.34	24.888	< 0.001		
HCY (*μ*mol/L) (mean ± SD)	Study group	17.17 ± 3.27	13.80 ± 2.85	25.668	< 0.001	2.524	0.014
	Control group	18.13 ± 3.69	15.60 ± 2.67	11.334	< 0.001		

Abbreviations: CRP, C‐reactive protein; HCY, homocysteine; IL‐6, Interleukin‐6; SD, standard deviation.

### 3.6. Vascular Function Indicators

As shown in Table 6, before treatment, there were no significant differences in ABI, TcPO_2_, or DPA flow velocity of the affected foot between the two groups (data not shown in the table, consistent with baseline comparability). After treatment, both groups showed significant improvements in these vascular function indicators compared with pretreatment (all *p* < 0.001). The study group had more obvious improvements: ABI (0.38 ± 0.07 vs. 0.20 ± 0.06, between‐group difference = 0.18, 95% CI: 0.15–0.21, Cohen′s *d* = 0.91, large effect), TcPO_2_ (18.67 ± 3.25 vs. 9.35 ± 2.78 mmHg, between‐group difference = 9.32 mmHg, 95% CI: 7.51–11.13, Cohen′s *d* = 0.87, large effect), and DPA flow velocity (0.18 ± 0.04 vs. 0.09 ± 0.03 m/s, between‐group difference = 0.09 m/s, 95% CI: 0.07–0.11, Cohen′s *d* = 0.83, large effect) (all *p* < 0.05).

**Table 6:** Pre‐ and posttreatment comparison of other laboratory parameters within and between the two groups.

**Item**	**Group**	**Before treatment**	**After treatment**	**Within-group comparison**		**Posttreatment between-group comparison**	
				**t** **-value**	**p** **value**	**t** **-value**	**p** **value**
ABI (mean ± SD)	Study group	0.49 ± 0.15	0.88 ± 0.10	40.303	< 0.001	3.941	< 0.001
	Control group	0.53 ± 0.08	0.76 ± 0.14	21.083	< 0.001		
TcPO_2_ (mmHg) (mean ± SD)	Study group	31.50 ± 5.85	51.57 ± 9.63	24.882	< 0.001	3.047	0.004
	Control group	32.70 ± 5.81	45.43 ± 5.37	36.852	< 0.001		
DPA flow velocity of affected foot (m/s) (mean ± SD)	Study group	0.17 ± 0.02	0.48 ± 0.04	120.882	< 0.001	14.411	< 0.001
	Control group	0.18 ± 0.03	0.30 ± 0.06	22.827			

Abbreviations: ABI, ankle–brachial index; DPA, dorsalis pedis artery; SD, standard deviation; TcPO_2_, transcutaneous partial pressure of oxygen.

### 3.7. Quality of Life and Patient Satisfaction

To further evaluate the clinical relevance of the intervention, assessments of quality of life (using the SF‐36) and patient satisfaction were added. As shown in Table 7, before treatment, there were no significant differences in all SF‐36 domain scores between the two groups (all *p* > 0.05). After treatment, both groups showed significant increases in PF, BP, GH, and SF domain scores compared with pretreatment (all *p* < 0.001), and the study group had significantly higher scores than the control group in all these domains (all *p* < 0.001). Before treatment, there was no significant difference in satisfaction score between the two groups (*p* > 0.05). After treatment, both groups showed significant increases in satisfaction score compared with pretreatment (all *p* < 0.001), and the study group satisfaction score (22.67 ± 1.89) was significantly higher than that of the control group (18.45 ± 2.12) (*t* = 12.345, *p* < 0.001).

**Table 7:** Pre‐ and posttreatment comparison of quality of life (SF‐36) and patient satisfaction between the two groups.

**Indicator**	**Group**	**Before treatment (** **m** **e** **a** **n** ± **S** **D** **)**	**After treatment (** **m** **e** **a** **n** ± **S** **D** **)**	**Within-group comparison (** **t** **-value,** **p** **value)**	**Posttreatment between-group comparison (** **t** **-value,** **p** **value)**
SF‐36 domain scores					
Physical functioning (PF)	Study group	45.23 ± 6.18	68.56 ± 7.24	*t* = 18.321, *p* < 0.001	*t* = 5.672, *p* < 0.001
	Control group	46.15 ± 5.89	55.32 ± 6.91	*t* = 9.453, *p* < 0.001	Control group
Bodily pain (BP)	Study group	38.76 ± 5.92	62.14 ± 6.58	*t* = 22.567, *p* < 0.001	*t* = 6.891, *p* < 0.001
	Control group	39.21 ± 5.76	50.45 ± 6.23	*t* = 11.234, *p* < 0.001	Control group
General health (GH)	Study group	42.35 ± 6.01	65.78 ± 7.12	*t* = 20.145, *p* < 0.001	*t* = 4.987, *p* < 0.001
	Control group	43.12 ± 5.98	56.89 ± 6.87	*t* = 10.876, *p* < 0.001	Control group
Social functioning (SF)	Study group	40.18 ± 5.87	63.45 ± 6.79	*t* = 19.872, *p* < 0.001	*t* = 5.234, *p* < 0.001
	Control group	41.05 ± 5.72	54.21 ± 6.53	*t* = 10.123, *p* < 0.001	Control group
Patient satisfaction score (total: 5–25)	Study group	12.35 ± 2.18	22.67 ± 1.89	*t* = 35.678, *p* < 0.001	*t* = 12.345, *p* < 0.001
	Control group	12.89 ± 2.05	18.45 ± 2.12	*t* = 18.901, *p* < 0.001	

### 3.8. Equivalence Testing Results

For the primary outcome ABI, the 90% CI of the between‐group difference (study group–control group) was 1.52–2.11, which exceeded the preset equivalence margin (*Δ* = 0.1), indicating that the combined therapy was not equivalent to exercise alone but superior in improving lower limb perfusion. For the VAS score, the 90% CI of the between‐group difference (reduction in study group–reduction in control group) was 1.32–2.02, exceeding *Δ* = 1.0, confirming that the combined therapy had a more significant pain‐relieving effect. For the Barthel index, the 90% CI of the between‐group difference (increase in study group–increase in control group) was 11.05–15.63, exceeding *Δ* = 10, further supporting the combined therapy′s superiority in enhancing daily living ability. No equivalence was observed for blood glucose, lipid, or inflammatory markers (all 90% CIs exceeded respective *Δ*), consistent with the between‐group difference results.

## 4. Discussion

LEAD is a serious chronic complication of DM, often leading to severe morbidity, including amputation due to diabetic foot ulcers. The prevalence of LEAD is high among patients with Type 2 DM, and it is associated with increased cardiovascular risk and mortality [35]. Given the high amputation rates and considerable impact on quality of life, the early detection and effective management of LEAD are crucial. In this study, patients with LEAD received the combination therapy of ASOT and upper and lower limb rehabilitation training on the basis of conventional treatment.

The changes in inflammatory markers observed in this study could potentially be influenced by the treatment of concurrent infections. However, several factors support the conclusion that the observed reductions in inflammatory markers were primarily due to the therapeutic interventions rather than merely reflecting the resolution of infection‐related inflammation. First, all patients underwent a thorough infection assessment upon admission and received targeted antibiotic therapy if necessary. The management of infections was part of the standard care and not directly related to the study interventions. Therefore, although infection control may have contributed to the initial reduction in inflammatory markers, the sustained improvements observed over the 10‐day treatment period suggest a broader effect of the combined therapy (ASOT and exercise) on systemic inflammation. Second, we measured inflammatory markers (IL‐6, CRP, and HCY) at multiple time points before and after treatment. The consistent and significant reductions in these markers across the study period indicate a sustained therapeutic effect rather than a transient response to infection resolution. The improvements were observed in both the control and study groups, with more pronounced effects in the study group receiving ASOT, further supporting the role of the intervention in modulating inflammation. Finally, our statistical analysis accounted for the presence of infection by stratifying patients based on their infection status. This approach allowed us to assess the independent effect of the treatment on inflammatory markers, independent of infection status. The results showed significant reductions in inflammatory markers in both groups, with the study group demonstrating superior outcomes, suggesting that the treatment had a substantial impact on inflammation beyond the management of concurrent infections.

### 4.1. Mechanistic Insights Into Therapeutic Effects

The findings of this study indicate that the combination of ASOT and rehabilitation training significantly improves pain symptoms, daily living abilities, metabolic control, and circulatory function in patients with DM‐LEAD. The key mechanisms underlying these effects are described below.

#### 4.1.1. ASOT‐Mediated Tissue Oxygenation and Angiogenesis

ASOT delivers high‐concentration oxygen under normobaric conditions, increasing PaO_2_ and improving oxygen supply to ischemic lower limb tissues [25]. This reduces tissue hypoxia, a key driver of LEAD progression [22]. Additionally, ASOT may activate Hypoxia‐Inducible Factor 1 (similar to HBOT [24]), stimulating the expression of vascular endothelial growth factor (VEGF) and other angiogenic factors. This promotes collateral vessel formation, enhancing limb perfusion (evidenced by increased ABI, TcPO_2_, and DPA flow velocity in the study group).

#### 4.1.2. Exercise‐Induced Improvements in Circulation and Metabolism

The MOTOmed‐based exercise training enhances muscle pump function in the lower limbs, improving venous return and arterial blood flow [16]. It also reduces blood glucose and lipid levels (by increasing insulin sensitivity [38] and lipid oxidation [39]), which alleviates endothelial dysfunction and atherosclerosis—key pathological processes in LEAD [17]. For example, the control group′s ABI increased by almost 0.4 after 10 days of exercise, likely due to these mechanisms.

#### 4.1.3. Synergistic Anti‐Inflammatory Effects

Both ASOT and exercise reduce systemic inflammation [22, 38]; ASOT inhibits the production of proinflammatory cytokines (e.g., IL‐6) by reducing tissue hypoxia [22], and exercise downregulates inflammatory pathways (e.g., the Toll‐Like Receptor 4 pathway [14]). The combination of these two therapies results in a more significant reduction in inflammatory markers (IL‐6, CRP, and HCY) in the study group, further slowing LEAD progression. This large effect size (Cohen′s *d* > 0.8) and narrow 95% CI (no overlap with 0) confirm the robustness of the anti‐inflammatory effect of the combined therapy.

### 4.2. Clinical Implications

The clinical implications of this integrated approach are threefold. First, this combined therapy offers a novel noninvasive treatment option for patients ineligible for revascularization or those with limited access to advanced medical resources, particularly those with advanced LEAD (Fontaine Stage IV) and severe comorbidities, who constituted the majority of the study cohort. Second, ASOT demonstrates superior cost‐effectiveness to HBOT, with improvements in ABI, TcPO_2_, and DPA flow velocity confirming its potential to delay disease progression and reduce amputation risks. Third, for the one‐third of patients with concurrent neuropathy, this regimen achieves dual management of vascular and neuropathic pathology by modulating inflammatory markers [26], blood glucose/lipid profiles, and oxidative stress—key contributors to the pathogenesis of DM‐associated LEAD. These results underscore the value of a multifactorial management strategy, providing evidence‐based support for comprehensive care in this high‐risk population.

One of the most notable findings in our study was the significant improvement in the ABI in the control group, which increased by almost 0.4 after just 10 days of exercise therapy. This improvement is remarkable given the short duration of the intervention and the advanced stage of the disease in our patient population. The underlying physiological mechanisms contributing to this improvement likely include enhanced blood flow dynamics, muscle pump mechanisms, a reduction in blood glucose and lipid levels, and neurovascular adaptations. Exercise therapy, particularly using the MOTOmed rehabilitation trainer, promotes increased blood flow through the affected limbs, leading to the recruitment of collateral vessels and improved perfusion, thereby enhancing the ABI [16]. Regular exercise strengthens the calf muscles, which act as a peripheral heart to aid in venous return and arterial flow, considerably improving blood flow dynamics in the lower extremities [17]. The exercise regimen likely contributes to improved metabolic control, with reductions in blood glucose and lipid levels, which can reduce endothelial dysfunction and inflammation, thereby enhancing vascular health [38]. Exercise‐induced improvements in nerve function and vascular reactivity can also play a role in enhancing blood flow and ABI, which is particularly relevant in patients with diabetes, where neuropathy and vascular disease often coexist [39].

The results showed that both groups experienced improved pain symptoms and daily living abilities, reduced blood glucose, blood lipid, and inflammatory marker levels, as well as increased ABI, TcPO_2_, and DPA flow velocity on the affected side. The addition of ASOT in the treatment group was associated with superior improvement in these parameters compared to the control group. There are several possible mechanisms underlying these improvements. Elevated levels of inflammatory markers represent an important mechanism contributing to LEAD [17]. Reducing these markers can clinically delay the progression of LEAD. In this study, the post‐ASOT levels of CRP, IL‐6, and HCY in the study group decreased significantly compared with the control group. High‐concentration oxygen therapy can increase PaO_2_, leading to improved tissue oxygenation and circulation [40]. In our study, ASOT resulted in significant improvements in TcPO_2_, ABI, and DPA flow velocity on the affected side. ASOT also plays a role in lowering blood glucose and lipid levels, which indirectly slows the progression of LEAD and reduces the amputation rate [25]. This may be explained by the ASOT‐mediated increase in cellular sensitivity to insulin and the consequent decline in blood glucose levels. Although ASOT features oxygen supply at high concentration levels, its oxygen pressure is lower than that of HBOT. Further studies are warranted to determine whether this affects treatment efficacy.

Despite advances in medical and surgical treatments, the management of DM and LEAD remains challenging. Current treatments often fail to adequately address the multifactorial nature of these conditions, leading to suboptimal outcomes. There is a considerable gap in the availability of noninvasive, cost‐effective, and accessible treatments that can effectively improve circulation, reduce inflammation, and enhance overall patient outcomes [6]. This gap is particularly evident in resource‐limited settings where advanced interventions such as HBOT are not readily available. The innovative combination of ASOT and rehabilitation training represents a key strength of this study. This approach addresses the limitations of current treatments by providing a noninvasive, accessible, and cost‐effective alternative. ASOT, by delivering high‐flow pure oxygen in a normobaric state, enhances tissue oxygenation and circulation, and rehabilitation training improves muscle strength and overall physical function. The combination of these therapies leverages their synergistic effects to achieve superior clinical outcomes.

### 4.3. Limitations and Future Directions

Although this study demonstrates the therapeutic potential of combining ASOT with rehabilitation training, several limitations must be acknowledged.

#### 4.3.1. Sample Size

The sample size (*n* = 60) is relatively small, which may limit the generalizability of the findings. However, the pre hoc power analysis confirmed that this sample size was sufficient to detect large‐to‐medium effect sizes (power = 0.85 for the primary outcome of ABI). Future studies with larger sample sizes (e.g., *n* > 100 per group) and multicenter designs are needed to validate these results in more diverse populations.

#### 4.3.2. Study Duration

The intervention duration (10 days) was short, and the follow‐up period was not extended beyond the treatment phase. Thus, the long‐term efficacy (e.g., 3–6 months after treatment) and safety (e.g., risk of oxygen‐related complications with prolonged use) of the combined therapy remain unknown. Short‐term improvements in surrogate outcomes (e.g., ABI and TcPO_2_) may not translate to long‐term reductions in amputation rates or mortality. Future studies should include extended follow‐up (e.g., 12 months) to assess sustained outcomes and long‐term safety.

#### 4.3.3. Blinding Limitations

Despite implementing measures to blind outcome assessors, treating physicians, and data analysts, complete blinding of patients and ASOT administrators was not possible (due to the visible use of the oxygen mask in the study group). This may have introduced performance bias (e.g., patients in the study group may have reported better pain relief due to expectations of treatment efficacy). Future studies could use a sham ASOT group (e.g., administering room air via the same mask) to achieve improved blinding.

#### 4.3.4. Patient Population

The study cohort primarily consisted of patients with advanced LEAD (Fontaine Stage IV, 75%), limiting the generalizability of the findings to patients with early‐stage LEAD (Stages I–III). Future trials should include patients with a broader range of LEAD stages to assess the preventive effects of the combined therapy in early disease.

#### 4.3.5. Outcome Measures

Although this study added patient‐reported outcomes (SF‐36, patient satisfaction), it still relies heavily on surrogate outcomes (e.g., ABI and inflammatory markers). Future studies should include hard clinical outcomes, such as amputation rates, cardiovascular events, and mortality, to better evaluate the clinical impact of the therapy.

#### 4.3.6. Mechanistic Studies

The study inferred mechanisms (e.g., angiogenesis and anti‐inflammatory effects) based on changes in surrogate markers but did not directly measure these mechanisms (e.g., VEGF levels and collateral vessel density via imaging). Future studies should include mechanistic analyses (e.g., serum VEGF measurement and contrast‐enhanced ultrasound for collateral vessels) to confirm the proposed therapeutic pathways.

## 5. Conclusion

This study provides evidence that ASOT combined with exercise therapy can substantially improve clinical outcomes in patients with DM and LEAD. The observed improvements in pain symptoms, daily living abilities, metabolic control, circulation, quality of life, and patient satisfaction highlight the potential of this combined therapeutic approach. Compared with international studies [9, 23, 37], this therapy offers a more accessible and cost‐effective option for DM‐LEAD management, particularly in resource‐limited settings. Future research should aim to address the limitations identified in this study (e.g., larger sample size, extended follow‐up, and mechanistic analyses) and further explore the clinical applications of ASOT in the management of LEAD.

## Ethics Statement

This study was conducted in accordance with the Declaration of Helsinki and approved by the ethics committee of the Second Hospital of Hebei Medical University (Approval No. 2024‐R255). We obtained signed informed consent from the participants in this study.

## Consent

The authors have nothing to report.

## Disclosure

All authors read and approved the final manuscript.

## Conflicts of Interest

The authors declare no conflicts of interest.

## Author Contributions

L.Z., Z‐B.L., L‐C.W., and H‐L.L. conceived of the study, and Y.L., L.Z., Q‐X.Z., and H‐L.Z. participated in its design and data analysis and statistics. All authors helped draft the manuscript. Z‐B.L. and L‐C.W. contributed equally to this study.

## Funding

The study was funded by the Medical Science Research Project of Hebei (202305233).

## Data Availability

All data generated or analyzed during this study are included in this article. Further enquiries can be directed to the corresponding author.
